# Modeling Data Journeys to Inform the Digital Transformation of Kidney Transplant Services: Observational Study

**DOI:** 10.2196/31825

**Published:** 2022-04-21

**Authors:** Videha Sharma, Iliada Eleftheriou, Sabine N van der Veer, Andrew Brass, Titus Augustine, John Ainsworth

**Affiliations:** 1 Centre for Health Informatics Division of Informatics, Imaging and Data Science The University of Manchester Manchester United Kingdom; 2 Department of Renal and Pancreatic Transplantation Manchester University National Health Service Foundation Trust Manchester United Kingdom; 3 Division of Diabetes, Endocrinology and Gastroenterology The University of Manchester Manchester United Kingdom

**Keywords:** digital transformation, health information exchange, interoperability, medical informatics, data journey modelling, kidney transplantation

## Abstract

**Background:**

Data journey modeling is a methodology used to establish a high-level overview of information technology (IT) infrastructure in health care systems. It allows a better understanding of sociotechnical barriers and thus informs meaningful digital transformation. Kidney transplantation is a complex clinical service involving multiple specialists and providers. The referral pathway for a transplant requires the centralization of patient data across multiple IT solutions and health care organizations. At present, there is a poor understanding of the role of IT in this process, specifically regarding the management of patient data, clinical communication, and workflow support.

**Objective:**

To apply data journey modeling to better understand interoperability, data access, and workflow requirements of a regional multicenter kidney transplant service.

**Methods:**

An incremental methodology was used to develop the data journey model. This included review of service documents, domain expert interviews, and iterative modeling sessions. Results were analyzed based on the LOAD (landscape, organizations, actors, and data) framework to provide a meaningful assessment of current data management challenges and inform ways for IT to overcome these challenges.

**Results:**

Results were presented as a diagram of the organizations (n=4), IT systems (n>9), actors (n>4), and data journeys (n=0) involved in the transplant referral pathway. The diagram revealed that all movement of data was dependent on actor interaction with IT systems and manual transcription of data into Microsoft Word (Microsoft, Inc) documents. Each actor had between 2 and 5 interactions with IT systems to capture all relevant data, a process that was reported to be time consuming and error prone. There was no interoperability within or across organizations, which led to delays as clinical teams manually transferred data, such as medical history and test results, via post or email.

**Conclusions:**

Overall, data journey modeling demonstrated that human actors, rather than IT systems, formed the central focus of data movement. The IT landscape did not complement this workflow and exerted a significant administrative burden on clinical teams. Based on this study, future solutions must consider regional interoperability and specialty-specific views of data to support multi-organizational clinical services such as transplantation.

## Introduction

Data journey modeling is an emerging methodology developed to help understand the sociotechnical challenges and boundaries of data movement as part of digital transformation [1,2]. It has been used successfully to identify risks and costs of information technology (IT) projects within health care systems, such as the United Kingdom National Health Service (NHS) [3]. Specifically, data journey modeling provides a high-level overview of data entities, IT systems, manual processes, and organizations associated with a clinical service. It is a cross-collaborative methodology bridging health informaticians and clinical domain experts with the aim of producing a conceptual overview of the IT infrastructure pertinent to a clinical service. This allows a better understanding of how services are delivered from a data-centric perspective and helps inform meaningful solutions. As such, data journey modeling has been shown to identify opportunities for improving operational efficiency, data management, and patient safety, among other potential benefits [3]. The purpose of this study was to apply data journey modeling to a specific clinical use case, kidney transplantation, that was planning to undergo digital transformation.

Kidney transplantation is a regional, multi-organizational clinical service [4]. It is delivered at large university hospitals (in transplant centers), which receive patients from neighboring renal referral units. This hub-and-spoke model allows a wide geographical area to be covered and is similar to other specialist services, such as cancer, genetics, and vascular services. The patient journey in transplantation is complex and requires the capture of large volumes of heterogeneous clinical data. Multiple clinical teams are involved, and patients naturally cross organizational boundaries as they transition from declining kidney function to kidney failure and ultimately to kidney transplantation. Data capture during this patient journey requires meticulous administration to prevent delays and bottlenecks [5]. However, managing high-volume, complex clinical data across organizations is time consuming and error prone and incurs significant administrative costs. The 2014 United Kingdom Transplant First initiative recognized this, singling out “inefficient use of technology and administrative support” as one of the key barriers to timely transplantation [6]. The *American Journal of Transplantation* further highlighted the impact of the lack of integration of hospital-wide electronic patient records (EPRs) on kidney transplant care [7].

Owing to the aforementioned reasons, transplantation is a clinical area that will benefit from digital solutions to improve the management and flow of data. Health IT has been shown to successfully achieve these intended benefits; however, novel interventions are often marred by non-adoption and failure. [8]. A lack of understanding of the technical and organizational context for change is one of the key factors limiting success [9,10]. Further barriers exist due to a lack of consideration of the social aspect of interventions, which rely on human input and are therefore affected by resistance to change and failure to share perceived benefits with end users [11]. New interventions are often developed without including end users in the requirement-gathering process, and as a result, solutions are unsuccessful at achieving their intended benefits [1,12]. In an effort to successfully overcome these challenges, data journey modeling was identified as a methodology to understand the current IT infrastructure and involve domain experts in developing potential solutions.

The transplant referral process is an integral part of the overall transplant patient journey. It depends on the capture of data from various internal and external sources at the transplant center, concluding with the patient being registered on the national organ waiting list. This study aims to understand this process from a data journey perspective. Specific objectives were to (1) map the data management processes, including the role of IT support in a regional transplant network, (2) identify challenges and categorize them based on established frameworks, and (3) use the resulting findings to suggest potential solutions.

## Methods

### Overview

We followed an iterative and incremental approach to build the data journey model with input from clinical and administrative domain experts. We used the modeling process to identify potential challenges to data management and validated the final version of the model with domain experts who were not involved in the original modeling.

### Context

The context for our study was the transplant center at the Manchester University NHS Foundation Trust (Manchester, United Kingdom). It is the largest kidney transplant center in the United Kingdom [13], receiving patients from 2 further regional renal referral units (Royal Salford NHS Foundation Trust and Lancashire Teaching Hospitals NHS Foundation Trust). The transplant center registers around 300 new patients on the national transplant waiting list every year. Patients are also under the care of a local general practice, which maintains long-term well-being through community-based medical care.

**Figure 1:**
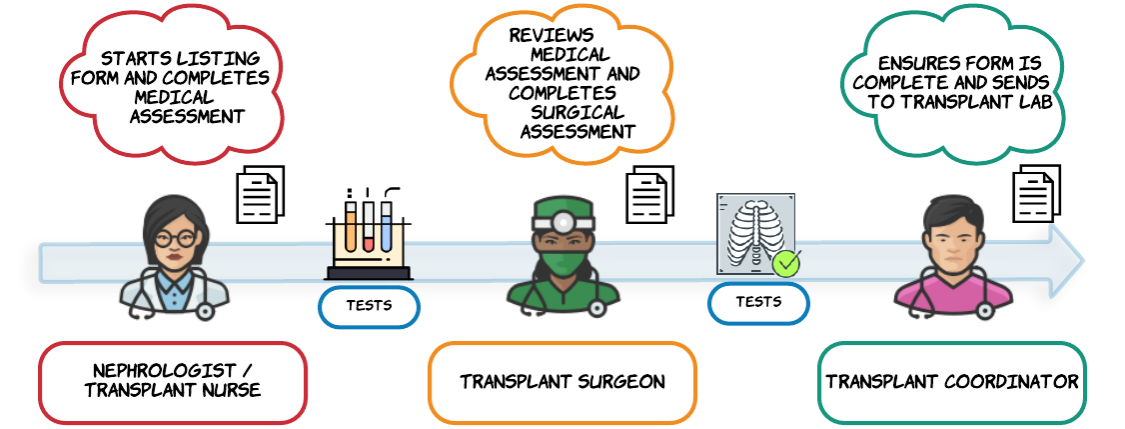
Data management in the transplant referral pathway is based on the transplant listing form.

Each referral includes several hospital visits, medical tests, and clinical assessments. Multiple health care professionals are involved at different stages of the pathway. Data capture along the pathway is undertaken on a Microsoft Word (Microsoft Inc) document called the “listing form.” Various sections of the listing form are populated with patient data by members of the clinical team at multiple clinical time points. The captured data include routine health care data, such as medical history, test results, and examination findings. A complete and accurate listing form is required to assess the fitness of patients for transplantation and to permit registration on the national waiting list. Once the form is completed and the patient is deemed suitable for transplantation, the form is sent to the transplantation laboratory for registration (Figure 1).

### Data Journey Modeling

**Figure 2:**
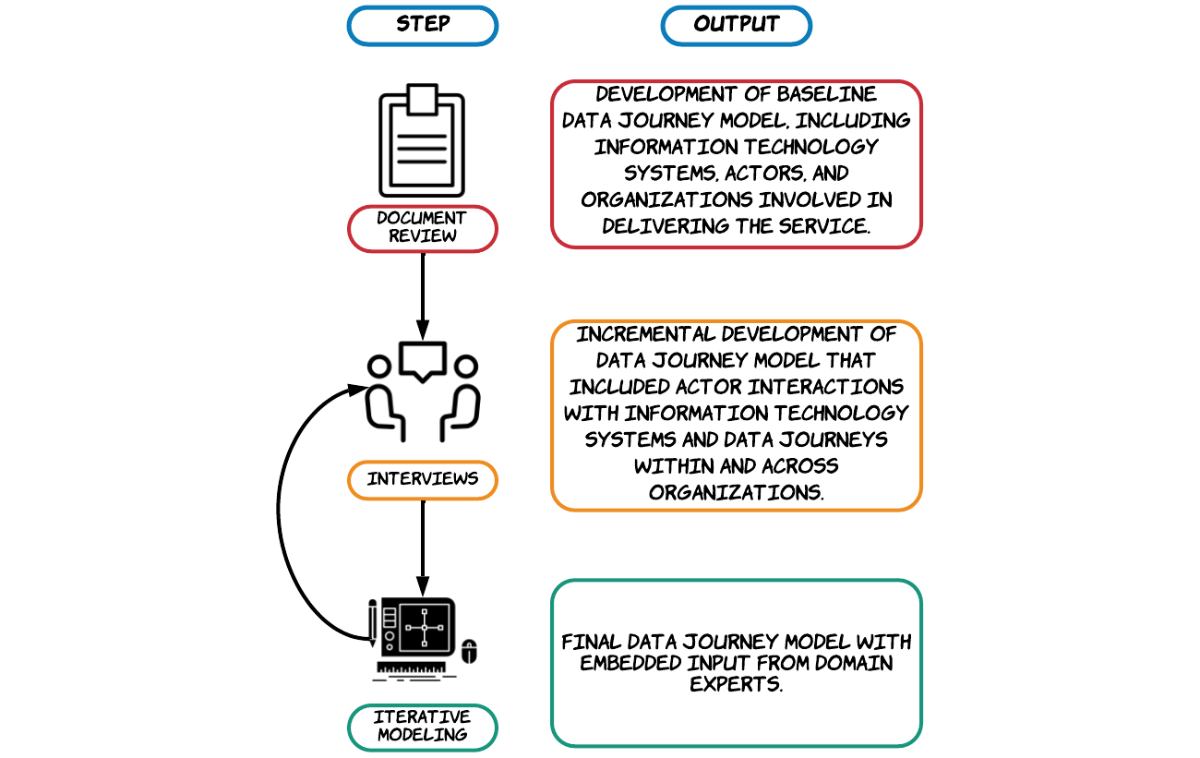
Summary of data journey modeling steps with associated output of each step.

Data journey modeling had 3 steps, summarized in Figure 2. The aim was to establish which IT systems contained transplant-related data, which organizations were involved in delivering the service, and which individuals delivered direct care or administration (ie, which individuals were actors), and to understand the interactions of the actors with the systems. This would provide a comprehensive overview of the IT infrastructure, the processes undertaken to extract and store data, and the data journeys, as part of the referral pathway. We then analyzed the results using an established framework, which was developed alongside data journey modeling, to help characterize our findings and draw meaningful conclusions [1]. Finally, we evaluated the final version of the model and our findings from the modeling process with domain experts who were not originally involved in developing the methodology.

### Document Review

We reviewed local written protocols pertaining to deceased donors, living donors, and transplant recipient pathways at the transplant center. We extracted all data entities routinely expected to be captured on the listing form and cross-referenced which IT systems these items were stored in. We identified which other health care organizations were involved in delivering the service and drew their boundaries. Finally, we established which actors played a role in the referral pathway within the transplant center. With this information, we designed a baseline iteration of the data journey model that demonstrated the technical and organizational infrastructure but was still missing the actors and data journeys. We used Lucidchart software (Lucid Software, Inc), a web-based diagram and visual design application, to draw our model iterations.

### Domain Expert Interviews

We conducted informal interviews and held small group meetings with domain experts working at the transplant center to gather information needed to further develop the model. We defined a domain expert as any member of the clinical or administrative team that was involved in direct patient care or back-office management of transplant-related data. We ensured this covered all the necessary actors identified through document review and the baseline iteration of the model. We spoke with 4 transplant coordinators, 2 nephrologists, 2 surgeons, 1 transplant assessment nurse, 2 secretaries, and 1 laboratory administrator. Domain expert interviews provided information on the processes used to extract and store data and the data journeys between IT systems and across organizational boundaries. Meetings lasted between 15 and 60 minutes; we kept written records of these meetings to increase accuracy and recall.

### Iterative Modeling

We followed an Agile-inspired method to develop the model, based on an incremental approach. Agile is an adaptive project methodology that relies on continuous collaboration with stakeholders to change the output based on feedback and repeated cycles of review [14,15]. It has been shown to successfully accomplish goals in health care projects and is well suited to the development of a model that depends on embedding feedback from domain experts to iterate a final version [16].

We modeled the processes that the various actors undertook in their work to deal with the key data entities and either capture and store or move data from one system to another. A total of 5 iteration sessions were held with the data journey modeler and domain experts to create the final model for analysis.

### Analysis and External Evaluation

**Figure 3:**
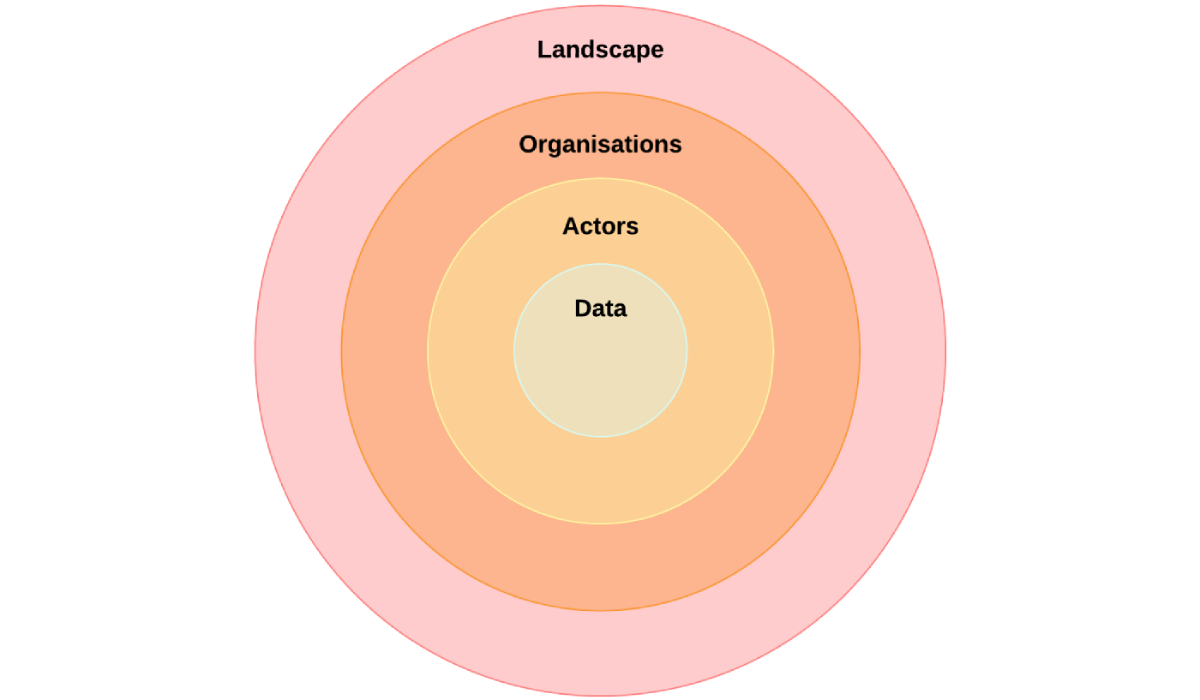
LOAD framework. LOAD: landscape, organizations, actors, data.

We used the LOAD framework to analyze the final version of the data journey model and categorize our findings. LOAD stands for “landscape, organization, actors, and data,” each denoting a dimension of IT as part of a clinical service (Figure 3) [1]. Using the LOAD framework ensured we comprehensively analyzed the model and associated data journeys, allowing us to identify technical barriers, such as lack of systems interoperability, and social challenges, such as manual work-arounds.

We then externally evaluated the final model by conducting semistructured interviews with domain experts who were not directly involved with model development. Interviewees included 2 transplant coordinators, 1 transplant surgeon, and 1 nephrologist. We presented them with the model and asked them if it accurately reflected the clinical workflow and data management processes at the transplant center. We prompted them to consider elements of the LOAD framework and think about how time spent on data management impacted delivery of the service and patient experience. The meetings typically lasted 30 minutes and were recorded in the form of research notes.

## Results

### Baseline Iteration of the Data Journey Model

Based on the document review, we established the basic elements of our model. There were four organizations contributing patient data pertinent to delivering the service: 1 transplant center, 2 referring centers, and 1 general practice clinic. Within the transplant center, we identified 6 IT systems that held data related to the transplant referral pathway (Table 1). There were also several external IT systems outside of the organizational boundary of the transplant center that contained pertinent data. These were systems at general practice clinics containing medical history and medication data and systems at other trusts containing local medical history and results. As we did not map IT systems at other organizations in detail, we denoted them as a single IT system, although each organization may have had multiple systems in use. Finally, once data collection along the clinical pathway was complete, the data were transferred through a web-based system called Organ Donation and Transplantation Online (developed in-house by NHS Blood and Transplant) to register the patient on the national waiting list.

**Figure 4:**
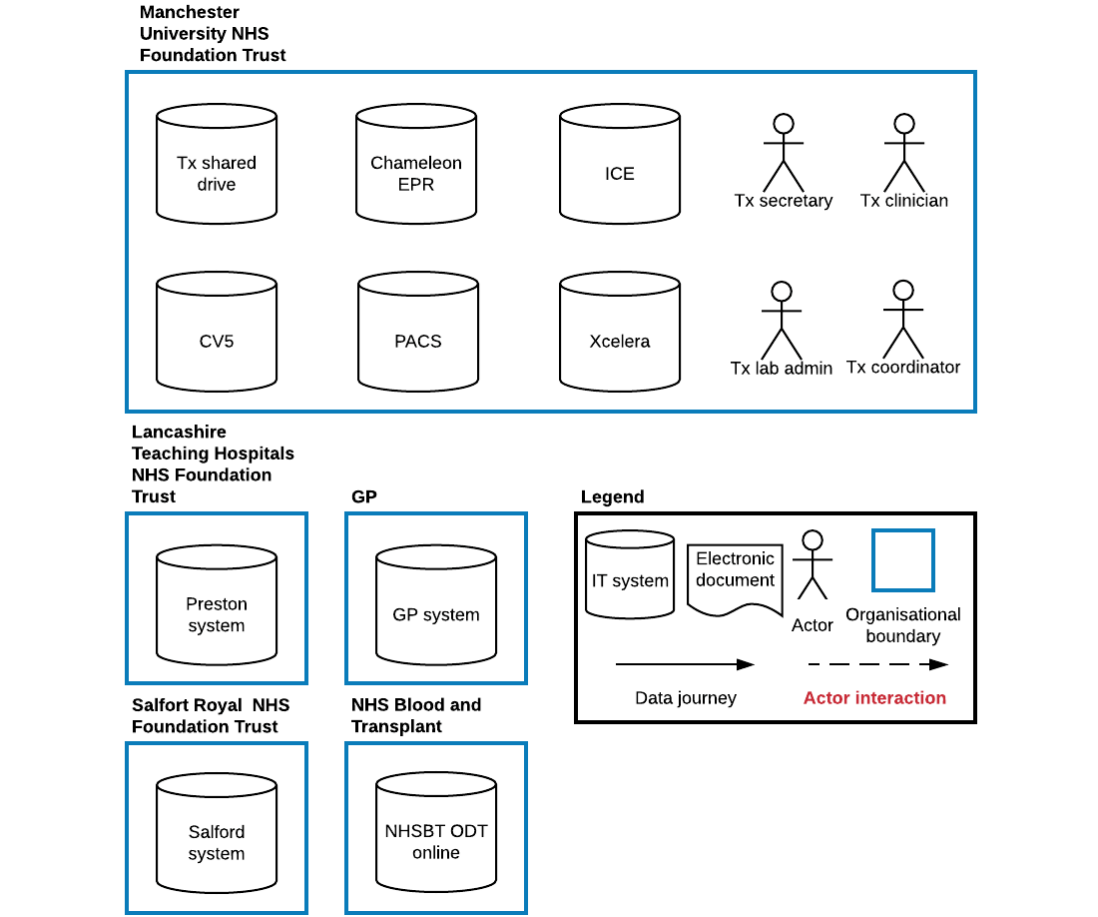
Baseline iteration of data journey model demonstrating information technology systems, organizational boundaries, and actors. CV5: Clinical Vision 5; EPR: electronic patient record; GP: general practice; ICE: integrated clinical environment; IT: information technology; NHS: National Health Service; NHSBT ODT: National Health Service Organ Donation and Transplantation; PACS: picture archiving and communication system; Tx: transplant.

We identified a total of 4 actors that played a role in managing clinical data: clinicians, transplant coordinators, secretaries, and administrators. The term “clinician” referred to multiple specialists, including nephrologists, surgeons, and transplant assessment nurses. However, as their roles were similar from a data perspective, we denoted them as “transplant clinicians” for the purposes of our model. Figure 4 demonstrates the output of document review and the first iteration of the data journey model.

**Table 1:** Summary of all information technology systems at the Manchester University NHS Foundation Trust, their suppliers, and their clinical data management purposes. NHS: National Health Service; EPR: electronic patient record.

System	Supplier	Purpose
Chameleon EPR	In-house	Correspondence/results
Integrated Clinical Environment	CliniSys Group	Ordering tests
Picture Archiving and Communication System	General Electric Co.	General radiology
ClinicalVision 5	Constellation Kidney Group	Renal history/dialysis details
xCELERA	Philips NV	Cardiovascular imaging
Shared drive	Microsoft Inc.	Transplant listing form

### Final Data Journey Model

**Figure 5:**
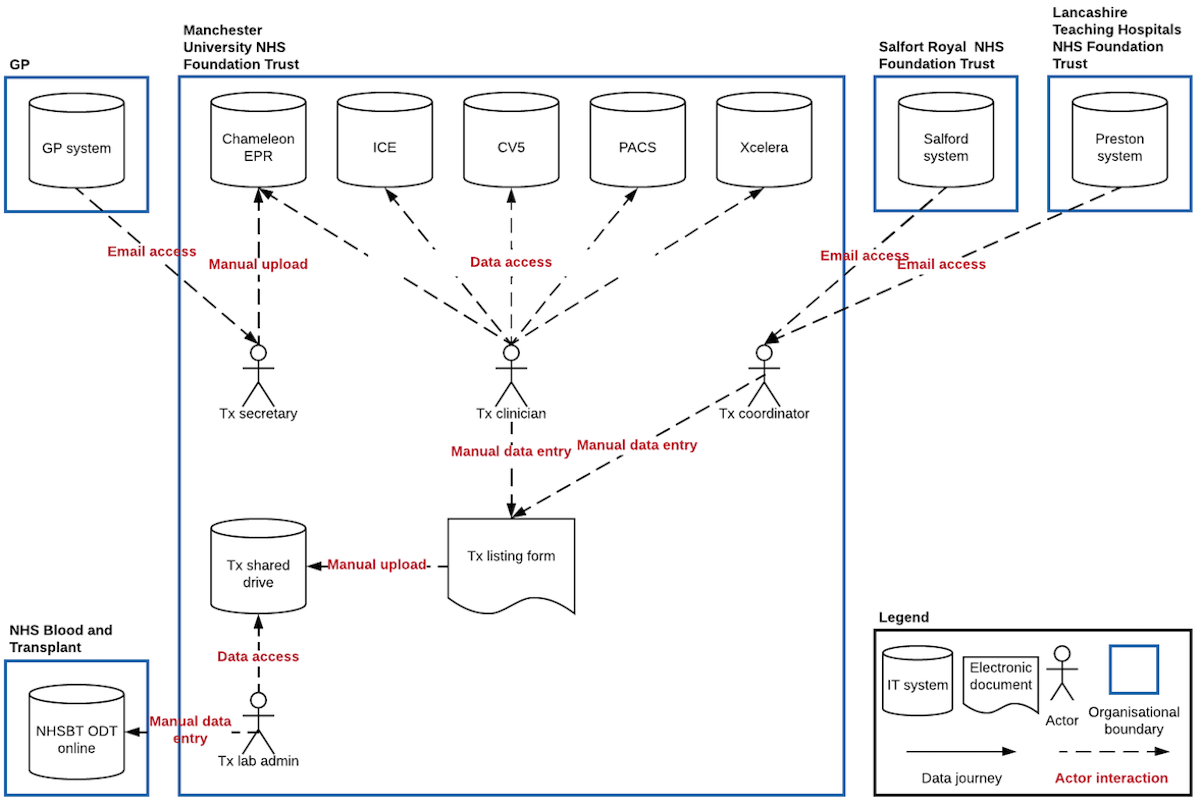
Final data journey model, demonstrating the information technology landscape and data journeys in kidney transplant referral. CV5: Clinical Vision 5; EPR: electronic patient record; GP: general practice; ICE: integrated clinical environment; IT: information technology; NHS: National Health Service; NHSBT ODT: National Health Service Organ Donation and Transplantation; PACS: picture archiving and communication system; Tx: transplant.

The baseline model and domain expert interviews iteratively informed actor interactions and data journeys, which were added to the model to create the final version. The organizations were rearranged, placing the transplant center at the center of the model and the other organizations around it. There were no direct data journeys between IT systems within the transplant center or between systems across organizational boundaries. It became clear that the shared drive was the central focus of data management. This was an in-house solution resulting from the need to centrally capture and view clinical data; this need was not being met by existing systems. To complete the workflow, a minimum of 12 separate actor interactions with IT systems were necessary. Actors had the following minimal number of interactions with the IT systems: clinicians, 5 interactions; coordinators, 3 interactions; secretaries, 2 interactions; and administrators, 2 interactions. The final data journey model is shown in Figure 5*.*

### LOAD Analysis

The final data journey model and feedback from external evaluation by domain experts allowed us to analyze findings based on the LOAD dimensions.

### Landscape

The overall landscape demonstrated the complexity of the transplant referral pathway from a data perspective. The IT systems were not developed for the needs of the transplant service and have not been updated as the requirements have changed over time. A lack of interoperability across organizational boundaries raised data governance issues, and it was unclear whether data sharing between the organizations were the result of formal agreements. There was no IT system that provided a unified view of transplant data, which resulted in a work-around solution in the form of Microsoft Word documents and shared drives. This has led to a landscape where human actors, rather than IT systems, form the central focus of data movement.

### Organizations

Key data were mainly stored internally within the transplant center’s organizational boundary. Patient data, such as results of investigations not undertaken at the center, were stored externally at referral units and general practices. There were no direct data journeys from IT systems at external organizations to the transplant center; this data was typically transferred via post, email, or fax to the transplant coordinators. They then manually scanned paper-based data and saved it to the shared drive alongside other electronic data. We found that two-thirds of the patients going through the pathway were from external referral units. This meant that for the majority of patients registered on the waiting list, there were no up-to-date clinical data at the transplant center. All interviewees reported that this posed a significant challenge to clinical workflow. Time was spent chasing down data from referral units and there were frequent delays due to the need for repeated requests. An additional social challenge was the lack of accountability, with clinical staff being unclear who was responsible for data being updated and accurate: the transplant center or the referral units.

### Actors

Data journeys were wholly dependent on actor interaction with IT systems and manual transcription of data. Key data was stored across multiple IT systems, which led to loss of efficiency as clinicians had to log in multiple times to view and extract data. Only 2 actor groups were able to interact with the shared drive, which meant that in their absence a patient would not be able to progress along the listing pathway. Domain experts reported that this created a bottleneck for the overall data journey and resulted in patient delays. Due to the impracticality of switching between multiple applications to access and transcribe data, actors reported using heuristic work-arounds, such as the use of 2 devices (eg, laptop and desktop). However, from interviews it emerged that there was variation in digital aptitude, and actors reported a range of experiences of interacting with the systems.

### Data

We found that the listing form included a total of 247 data fields that needed to be populated. All required data were stored in the 5 IT systems of the transplant center and in the systems of the general practice or referral centers. There were no data journeys between IT systems or from systems to the transplant shared drive. To move data to the shared drive, clinicians had to access the different systems and transcribe (ie, type) clinical data into the relevant fields and save the form in the designated shared folder. The file name was saved as the patient’s first and last name. All data required to populate the form were in electronic format. Data were directly transcribed without any clinical expertise required for transformation or manipulation. Domain expert interviews revealed that transcription errors and incomplete data fields were a source of both patient risk and delays in the listing pathway. There was also no current way of confirming data accuracy or obliging data completion. Interviewees further expressed their frustration at the time-consuming nature of the tasks, which detracted from time spent with patients.

### Risk Mitigation Strategies

The above findings suggest that a regional solution with an agreed data sharing and governance contract would help mitigate the risks of the current fragmented landscape. A need has emerged for a central clinical data repository with a user interface accessible at the transplant center and referral units. Considering the range of multi-disciplinary actors involved in the transplant referral pathway, the user interface will have to be adaptable and easy to operate in order to lower barriers to adoption. Technically, such a solution would benefit from being web based and from using cloud storage to provide security and safe access across organizational boundaries. Interoperability and open data standards would underpin this integration of data across IT systems. Critically, a deep understanding of needs and requirements, as provided through the results of this study, should drive the development of solutions to achieve the intended benefits. This also holds true for health IT projects in other clinical domains, demonstrating the value of this methodology.

## Discussion

### Principal Findings

This study applied data journey modeling to evaluate the kidney transplant referral pathway and successfully identified the data, IT systems, actors, and organizations involved, as well as the relationship between them. This has provided an overview of the data landscape and highlighted the complexity of data administration, as well as the lack of data flow. We found that clinical staff must undertake cumbersome manual processes to summarize and visualize data from multiple IT systems. Work-arounds have been created in the absence of a meaningful solution to address the needs and requirements of the clinical workflow. The lack of interoperability and central access to relevant data increases the effort and time required to complete transplant referral, which can delay patients’ registration on the transplant list.

### Relation to Other Studies

This is the first study to apply data journey modeling to transplant services. Previous studies have highlighted the complexity of kidney transplantation from a clinical management perspective. These recommended the use of IT solutions, such as business process management technology, to lower management costs [17]. Our study has established the dependence on manual processes to administer data, which is likely to incur management costs. The current data landscape strictly serves a documentation process, and does not provide any process support. Experience across the European Union shows that contemporary IT systems and EPR systems must provide functionality beyond data capture to better support the needs of clinical services [18].

This study found that data journeys in the transplant pathway naturally crossed specialty and organizational boundaries. However, with the absence of interoperability there was a dependence on actor interaction to share data. In other clinical areas, access to data across organizational boundaries continues to be a significant challenge [19]. The introduction of a national EPR system in Finland has facilitated implementation of digital pathways across nephrology and transplantation [20]. However, larger nations with more heterogeneous populations and geographical variations face challenges in harmonizing fragmented health care data [21]. Data journey modeling, such as that performed in this study, confirms that interoperability remains one of the key barriers to meaningful digital transformation.

### Implications for Practice and Future Concepts

Data journey modeling showed that during the referral pathway, clinicians are not required to transform or manipulate any data in order to complete the form—thus the IT challenge is one of summarizing and viewing relevant information in a format that allows seamless and enhanced clinical decision-making. In the United Kingdom, general practices recognize the value of early, customized viewing of clinical data, and primary-care IT systems are more intuitive for clinicians’ use [22]. However, in the hospital setting, a paradox exists in which IT systems commonly detract from patient contact due to dependence on user interaction to view data [23,24]. An early study by Zeng et al [25] evaluated concept-oriented views of clinical data versus traditional chronological presentation of data, such as in current EPRs. They demonstrated that visualizing data in their clinical context, such as the disease or organ system, reduced information overload and increased the accuracy of data retrieval. Based on the data fields identified in this study, for kidney transplantation this would include presenting a single-screen summary of relevant demographics, as well as medical and social history, with details pertinent to dialysis and previous surgeries. This would allow clinicians to focus on the patient at the time of an encounter, and add relevant clinical details not previously recorded in any IT system to the patients' record, such as residual urine output, exercise tolerance, or examination findings [26].

**Figure 6:**
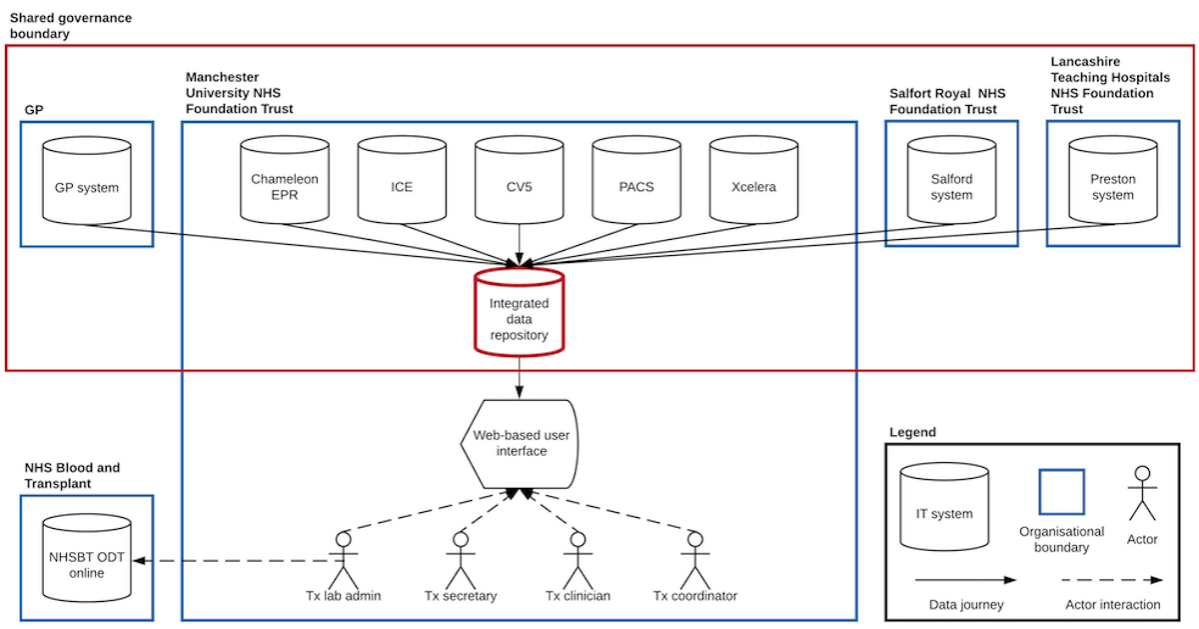
Conceptual overview of a proposed solution including a regional integrated data repository with a web-based clinical user interface. CV5: Clinical Vision 5; EPR: electronic patient record; GP: general practice; ICE: integrated clinical environment; IT: information technology; NHS: National Health Service; NHSBT ODT: National Health Service Organ Donation and Transplantation; PACS: picture archiving and communication system; Tx: transplant.

Findings from this study highlight the technical requirements for a transplant-specific solution with a regional, integrated data store that spans the relevant organizations with an application processing interface that meets the needs and requirements of the clinical workflow (Figure 6). Separate data and application layers for health care IT may help overcome current interoperability barriers and enable development of modular service-specific solutions [27]. Centralized clinical data repositories may facilitate application of model-view-controller software development, giving individual clinical areas the opportunity to design views to suit their context [28]. Semantic interoperability across systems allows data to be readily exchanged, analyzed, and interpreted, and is a prerequisite for meaningful digital transformation. In contrast, digital data stored in isolated databases not only slows down medical progress, but also limits technological innovations such as real time analytics and the reuse of data for research. [19,26]. Solutions such as Fast Healthcare Interoperability Resources protocols and OpenEHR archetypes may address these challenges going forward, but development is still required before widespread adoption [29,30].

The Healthcare Information and Management Systems Society has defined digital maturity of individual health care providers based on capabilities, interoperability, and governance [31]. However, due to the multicenter nature of transplant services, we found that digital maturity was limited by the least mature organization that formed part of delivering the service. Thus, even if the transplant center had an advanced and unified EPR system, the fact that patients were referred by other organizations unable to share data implied that clinical processes could not be adequately supported. Evaluating the potential impact of any novel solution should therefore be undertaken using interoperability frameworks [32]. In addition, capturing quantitative data, such as the time taken to be added to the transplant waiting list, could provide a measure of impact.

Across health care, clinical data remains constrained to organizational boundaries, and new EPR procurement does not actively consider regional workflow or data sharing, reinforcing vendor lock-in [33]. In response to this, NHS England launched the “Local Health and Care Record Exemplars,” a project tasked with increasing clinical information sharing across primary, secondary, and social care within a region [34,35]. Transplantation may be an excellent use case for such interoperability initiatives to demonstrate value to clinicians, policy-makers and, crucially, patients. Linked data will provide the basis for learning health systems that are intuitive to their populations’ needs and inform timely interventions to improve long-term health and social care outcomes. [36,37].

### Limitations of This Study

A number of other models to evaluate health IT infrastructure exist. The data journey model and LOAD framework have been developed based on the UK health care context and were chosen as the most appropriate tools to use [38]. However, they have not been widely applied in other clinical areas, potentially because they rely heavily on domain expertise to provide input during the modeling process. In our case, the study was led by a clinical research fellow who was able to help bridge the gap between the clinical and academic stakeholders. Finally, this study looked at only a single regional transplant center. This leaves it unknown to what extent our findings would translate to other regions, warranting further investigation.

### Conclusion

Complex clinical care pathways must be fully understood to allow meaningful solutions to be presented as part of digital transformation initiatives. Data journey modeling successfully provided valuable sociotechnical factors for health IT in kidney transplantation. It highlighted how a lack of interoperability led to time-consuming manual interaction with multiple systems to summarize data for transplant referral. Data crossed multiple organizational boundaries, and all movement of data depended on actor interaction, even though no data were transformed or manipulated. Future solutions must consider regional interoperability, bespoke systems that meet clinical requirements, and automated processes that free clinical staff from administrative burdens.

## Abbreviations

EHR: electronic patient record
GP: general practice
IT: information technology
LOAD: landscape, organizations, actors, data
NHS: National Health Service
